# Evaluation of Bone Age in Children: A Mini-Review

**DOI:** 10.3389/fped.2021.580314

**Published:** 2021-03-12

**Authors:** Federica Cavallo, Angelika Mohn, Francesco Chiarelli, Cosimo Giannini

**Affiliations:** Department of Pediatrics, University of Chieti-Pescara, Chieti, Italy

**Keywords:** skeletal development, height, X ray, children, bone age

## Abstract

Bone age represents a common index utilized in pediatric radiology and endocrinology departments worldwide for the definition of skeletal maturity for medical and non-medical purpose. It is defined by the age expressed in years that corresponds to the level of maturation of bones. Although several bones have been studied to better define bone age, the hand and wrist X-rays are the most used images. In fact, the images obtained by hand and wrist X-ray reflect the maturity of different types of bones of the skeletal segment evaluated. This information, associated to the characterization of the shape and changes of bone components configuration, represent an important factor of the biological maturation process of a subject. Bone age may be affected by several factors, including gender, nutrition, as well as metabolic, genetic, and social factors and either acute and chronic pathologies especially hormone alteration. As well several differences can be characterized according to the numerous standardized methods developed over the past decades. Therefore, the complete characterization of the main methods and procedure available and particularly of all their advantages and disadvantages need to be known in order to properly utilized this information for all its medical and non-medical main fields of application.

## Introduction

Evaluation of skeletal maturity is a common procedure frequently performed in clinical practice. Hand and wrist X-rays are considered as an important indicator of children's biological age. Nowadays, many methods are available to evaluate bone age. The images obtained by hand and wrist X-ray reflect the maturity of different bones. This information, associated with the characterization of the shape and changes of bones, represents an important factor of the biological maturation process. During growth, biological maturity is defined by several parameters, including the characterization of skeletal maturity, sexual maturity, dental elements eruption, menarche, spermarche, deepening of the voice, growth spurt, and the achievement of 95% of the adult height (1–3). Many of these parameters, and particularly growth spurt and menarche, correlate better with bone age compared to chronological age (4). Therefore, chronological age differs from bone age, so the two indexes need to be distinguished: chronological age is defined as the age in years between birth and the evaluation of a subject; bone age is defined by the age expressed in years that corresponds to the level of maturation of bones. This determination is based on the presence of particular centers of bone formation as well as the dimension and structure of the bones (3, 5–8). Bone age may be affected by several factors, including gender, nutrition, as well as metabolic, genetic, and social factors and either acute or chronic diseases, including endocrine dysfunction (3–9).

## Methods

A systematic search has been performed in PubMed to identify randomized controlled trials (RCTs), meta-analyses, and retrospective and prospective studies of different methods to evaluate bone age, focusing on strengths and weaknesses of each procedure. The keywords for the research have been “bone age” and “skeletal maturation.”

## Fields of Application of Bone Age

Bone age is an interpretation of skeletal maturity. The determination of bone age is important to properly assess and guide the evaluation of short or tall stature, impaired or accelerated growth, and delayed or early puberty (10). However, data obtained from the assessment of bone age can be widely used both in medical or nonmedical settings.

### Applications in Medical Field: Role of Delayed and Advanced Bone Age

By evaluating the data obtained from bone age in the clinical setting, it is possible to distinguish three main groups of subjects: patients with delayed bone age, patients with bone age appropriate to chronological age, and patients with advanced bone age (3, 8–10).

Constitutional delay of growth and puberty is one of the most common causes of delayed bone age (10). Conventionally, this clinical condition is defined by the presence of delayed bone age (at least 2 SD) compared to chronological age associated with short stature, a delay in both pubertal maturation, as well as in the achievement of adult height, compared to peers. These data distinguish these patients from patients with familial short stature, in whom bone age corresponds approximately to chronological age (11–14).

It is also possible to evaluate a physiological variant of familial early puberty (14), especially in some ethnic groups (15, 16).

Bone age may be used either in normal variants of delayed growth patterns with delayed puberty and accelerated growth patterns with early puberty, where it may be more consistent with height age and adult height prediction may be more consistent with genetics. Assessment of bone age is also important for the correct diagnosis, particularly with the aim of detecting the causes of bone age alteration including mainly endocrine and nutritional causes and chronic nonendocrine disorders and syndromes.

#### Endocrine and Nonendocrine Causes

Several endocrine diseases might induce changes in bone age (10). In particular, subjects with severe hypothyroidism may have a delayed bone age. Congenital hypothyroidism leads to growth arrest, delayed bone age, and short stature at birth. Ossification centers are defective, appearing in an irregular and mottled pattern, with multiple foci that coalesce to give a porous or fragmented appearance. These characteristics are mainly documented in large cartilaginous centers, such as the head of the femur, head of humerus, and the tarsal navicular bone and are known as stippled epiphyseal dysgenesis. When hypothyroidism is acquired during growth, secondary centers of ossification are predominantly affected, with delayed fusion of epiphysis and with an irregular and heterogeneous ossification. The metaphyseal end of long bones usually has a sclerotic band (17–19). In addition, subjects with long-lasting and untreated growth hormone (GH) deficiency have a delay in bone maturation. As well, hypophyseal alterations secondary to malformation, tumor, or infiltrative pathologies may also be associated with bone age delay consequently to a secondary GH deficiency or hypothyroidism. The presence of hypogonadism with the consequent lack of circulating estrogens, androgens, and other pubertal hormones may cause an important delay in bone maturation during pubertal period (20–25).

A delayed bone age is common in malnourished conditions associated with chronic diseases such as intestinal inflammatory chronic diseases, celiac disease, and cystic fibrosis (26–29).

It is also common in chronic inflammatory states or infectious diseases, such as juvenile idiopathic arthritis and states of immunodeficiency (30–37). Children with cardiac diseases, or those with chronic kidney or liver disease, may experience a delay in skeletal maturation (38–42).

In some psychiatric conditions, such as anorexia and in subjects with states of psychosocial stress or abuse, the presence of delayed skeletal maturation is documented (43–45). Bone age delay is also associated with genetic syndromes such as trisomy 21, Turner syndrome, and Russell–Silver syndrome (10, 46–48).

In premature babies, there is often a delayed skeletal maturation (49).

By contrast, subjects with hyperthyroidism may present precocious puberty associated with advanced bone age (18, 19). Moreover, weight gain and obesity are one of the most important causes of pediatric advanced bone age; the mechanisms underlying these alterations are not fully clarified, although insulin resistance and hormonal factors produced by adipose tissue might play an important role (50, 51). Likewise, some pathological clinical diseases such as ovarian tumors, Leydig cells or germ cells, as well as adrenal tumors or adrenal diseases (e.g., congenital adrenal hyperplasia) (52–55) are typically associated with excessive production of pubertal hormones that cause a rapid progression of bone age, thus advanced bone maturation.

Medical treatment, either oral or dermal, can affect pubertal effects on bone age. In particular, estrogens and oral contraceptives or creams containing testosterone or estrogens can cause an early closure of the growth plate. Similarly, an excessive intake of foods containing phytoestrogens such as soya, according to some studies, may have an effect on bone age progression (56–63).

### Nonmedical Field

Data obtained by hand and wrist radiography during bone age assessment are also used in many nonmedical fields for example in sports (64) and for national policy in many countries (10). In fact, bone age can provide important information for athletes in order to distribute physical, human, and monetary resources properly (65–67).

Assessment of bone age is often required during international immigration programs (68, 69). In many European countries, the increase in illegal immigration and above all the immigration of children and adolescents unaccompanied by parents and without identity documents posed important doubts and stressed the need for new procedures aimed at ensuring a better assistance and protection for young people. In Sweden, many asylum applications in 2016 were made by lone refugee children, thus requiring novel proposed guidelines. In particular, the method of medical age assessment proposed consisted of taking X-rays of wisdom teeth and MRI scans of knee joints, which are then analyzed by dentists and radiologists. In Italy, a multidisciplinary approach is suggested to evaluate bone age using the Greulich–Pyle TW3 methods for a complete characterization of chronological age of the refugee. However, bone age itself cannot be considered the only absolute and incontrovertible datum to define the chronological age (68–79); therefore, limits and accuracy of this examination in predicting chronological age, especially in relation to different ethnic groups and underlying diseases, need to be considered.

## Maturation and Skeletal Development

Skeletal maturation is based on the activation and interaction of a complex series of physiological mechanisms. This process is characterized by a predictable sequence of development and progression of ossification centers.

Each bone segment begins its maturation first in the primary ossification center and then, through different stages of enlargement and remodeling, reaches the final shape; many bones, like long bones, have many centers of maturation (epiphysis). Although several body areas have been studied over the years in order to define a standardized and universal method (3, 6), the wrist and knee areas represent the gold standard procedures (3, 7). In addition, studies have shown that, in some bones, ossification typically begins at birth, while in others, it typically begins between 14 and 17 years of life. Therefore, the bone maturation process can be better characterized by the evaluation of the knee region in children under the age of 3, while in those older than 3 years, the assessment of hand and wrist bones is the most appropriate (80–82).

At birth, long bones present different centers of ossification that proliferate continuously until the terminal or epiphyseal part of the bone melt definitively with the diaphyseal one. This process is strongly affected by numerous factors, including GH and insulin-like growth factor-1 (IGF-1). Moreover, a deficit of thyroid hormones or an excess of corticosteroids causes a cell division reduction in the proliferation zone, inducing a growth delay. Not only hormones but also gender might affect this process. In particular, bone age is more advanced in female than in male individuals with the same chronological age. In fact, the bone maturation process lasts longer in male than in female individuals (83–85), and the moment of closure of the epiphyseal region occurs is roughly 2 years earlier in girls than in boys. Therefore, carpal bones are not ossified at birth, and this process typically advances from the center of ossification (80). Usually, the first ossification center to appear is in the context of capitate and hamate at the second month in female individuals and around the fourth month in male individuals and remain the only useful observable features for the next 6 months.

**Figure 1:**
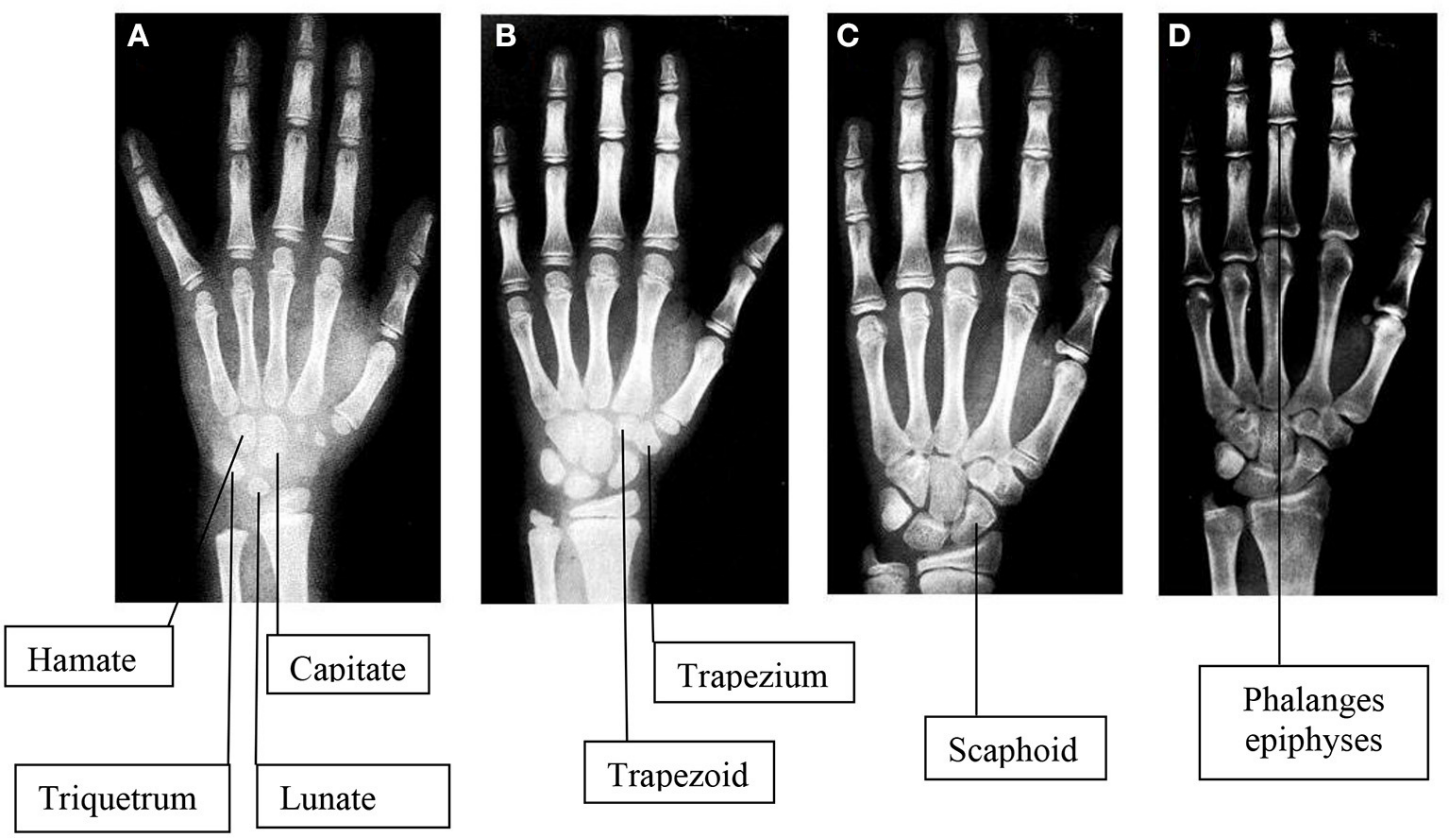
Images of hand and wrist x-rays in four female subjects compatible with physiological skeletal maturation in different ages: **A** (4 years), **B** (8 years), **C** (12 years), **D** (16 years). To note, usually the first ossification center to appear is in the contest of Capitate and Hamate at the second month in females and around the fourth month in males. Then the remaining centers appear, including Triquetrum at 2 years in females and 3 years in males, Lunate at 3 years in females and 4 years in males, Trapezium at 3 years in females and 4 years in males, Trapezoid at 4 years in females and 6 years in males, Scaphoid at 4 years in females and 6 years in males, Pisiform at 9 years in females and 12 years in males [77].

Then, the remaining centers progressively appear (Figure 1) (80). Assessments of skeletal maturity in prepubertal children are primarily based on the epiphyseal size of the phalanges as they relate to the adjacent metaphyses. During this stage of development, the ossification centers for the epiphyses increase in width and thickness, becoming as wide as the metaphyses. During puberty, the contours of the epiphyses begin to overlap, or cap, the metaphyses. Thereafter, the pisiform and the sesamoid become recognizable. During late puberty, the fusion of the epiphyses to the metaphyses in the long bones of the hand tends to occur in a characteristic pattern:

1. fusion of the distal phalanges,
2. fusion of the metacarpals,
3. fusion of the proximal phalanges, and
4. fusion of the middle phalanges.

After puberty, all carpals, metacarpals, and phalanges are completely developed, their physes are closed, and the assessment of skeletal maturity is based on the degree of epiphyseal fusion of the ulna and radius (80–82).

## Standardization of Hand and Wrist Radiography

During a hand and wrist X-ray procedure, the child is exposed to <0.00012 mSv of radiation, thus lower than other daily physiological risk (86), however resulting in irrelevant relative risk of 40-year mortality equal to 5.1 × 10^−8^ (calculated for an exposure dose of 0.00015 mSv) (87–89). For a correct interpretation of data, it is important to follow a correct procedure: the hand (conventionally the left hand) is positioned with the palm side resting on a rigid plane with fingers spread out to obtain an antero-posterior radiograph. The choice of the left hand depends on the fact that, at the time of sampling, the left hand was the less frequently impaired (at that time, many boys used to work in factories, and they could have suffered accidents at work). The middle finger axis should be in line with the forearm axis, and the center of the X-ray tube should be over the distal end of the third metacarpus. A distance of 76 cm from the box tube is recommend. The fingers are spread out so that they are not close together and the thumb is rotated in a natural position about 30° from the first finger. X-rays should be perpendicular to the rigid plane and usually be performed with a tube of 45–60 kVp potential.

## Assessment Methods for Evaluating Bone Age

Over the years, numerous standardized methods have been developed to evaluate a skeletal maturity score for the hand and wrist radiographs (3, 5, 90–92). Among these, three methods were the most representative and used worldwide: The Greulich–Pyle method, the Tanner–Whitehouse, and the Fels method. These different methods are characterized by a significant and variable intraindividual variability with values ranging from 0.96 to 0.74 years, which seems to be strongly affected by several factors including ethnicity, gender, and pubertal age (93–105).

### The Greulich and Pyle Method

Between 1931 and 1942, Greulich and Pyle evaluated the hand and wrist radiographs obtained from about 1,000 white people of Cleveland (Ohio, USA) belonging to medium–high social classes (9, 10, 106). Greulich–Pyle distinguished two standard templates: 31 and 27 radiographic images, in male and female individuals, respectively, which illustrate different phases of bone maturation between 0 and 18 or 19 years, respectively. Thus, gender-specific images are compared with the one obtained by patients by evaluating first the nearest chronological age and subsequently the adjacent standards. Therefore, during the procedure, the standard that seems similar is initially chosen, and then, the examination of each bone segment in an ordered sequence is performed by assigning the corresponding bone age to the individual segments, according to the instructions contained in the atlas text. It is important that not a simple comparison but an in-depth bone-by-bone evaluation is needed in order to properly characterized bone maturation. In fact, if a simple comparison is made, it is likely inadequate when the proximal and distal bones vary in maturation, sometimes by several years.

This method is very simple and fast, needing roughly 1.4 min for the evaluation (10, 107), thus explaining why it is preferred by 76% of pediatric endocrinologists and radiologists (10). The inability to be applied in children younger than 6 years or to perfectly match (equal to 100%) the images or to weigh the differences between bone structures (short and long) represents the main disadvantages of the procedure. The Greulich–Pyle tables can be applied in subjects belonging to Australia and the Middle East (108–110) but not to African or Asian populations (106, 111, 112).

### The Tanner–Whitehouse Method

The Tanner–Whitehouse method was developed in 1,930 using data obtained in European children (3, 113). It is based on the determination of a score obtained from hand and wrist skeletal maturation. The main advantage of this procedure related to the evaluation of each bone segment, thus minimizing the interoperator variability. Each of the bones that is evaluated is compared to a standard set of bones at different stages of maturation. A score is assigned to each bone based on maturation and sex of the patient. In this way, a maturity score is obtained for each area of clinical interest, generally categorized as A, B, C, D, E, F, G, H, and I. A numerical value is then assigned to each stage with specific differences between gender. This evaluation is more detailed than a simple comparison and takes into consideration a detailed analysis of structural characteristics of different bones with the assignment of a score to each element (3, 113, 114).

Over the years, this system has been refinished by moving from an initial system known as Tanner–Whitehouse method 1 (TW1) to two subsequent methods known as Tanner–Whitehouse 2 (TW2) and 3 (TW3) (3, 113, 114). Therefore, while in the TW1 version, the score is derived from the evaluation of all the 20 bones selected, in the TW2 update, three different ways are distinguished: “20 bones” score (as in TW1), “RUS” score (radius, ulna, and metacarpal bones and phalanx), and “CARPAL,” limited to carpus bones. The next version (TW3) takes into consideration only RUS bones, and it can be used through a software. Reference standards for this method were published in 1950 and 1960; however, from these initial publications, several studies have shown a shift toward an earlier bone maturation process in the general population worldwide (16). In order to improve the accuracy and reproducibility of this method, changes and improvements have been made over the years. Particularly, in the TW3, the possibility to predict final height has been introduced. Moreover, the score based on 20 bone segments was abolished, and the reference values and the graphs were modified and based on data obtained from native North American children. Numerous scales have been produced that can convert bone maturity score into bone age for different European and non-European populations (7, 114–119). Data described in the TW3 method show a progression of bone age typically between 10 and 12 years compared to that reported in the TW2 method; in particular, TW3 estimates of bone age are younger than TW2 especially in children with idiopathic short stature and constitutional delay in growth and puberty. This difference is less evident or absent for younger groups. For this reason, in the TW3 method, skeletal age evaluation ends at 15 years in women and 16.5 years in men (while in the TW2 set, 18 and 19 years, respectively, with a bone maturity anticipation of 2.5–3 years) (120). Tanner–Whitehouse method is more complex and time consuming, requiring approximately 7.9 min if the TW2 (121) method is used. This method has the advantage of being more reproducible, and it is not based on the subject age but on skeletal maturity of several bone elements and population-based references. If bone age reading is performed with the Tanner–Whitehouse method, there are some equations proposed by Tanner to calculate growth prognosis (10, 122, 123).

### The Fels Method

The Fels method was developed by Roche through a longitudinal study, based on a total of 13,823 serial X-rays of the left hand and wrist. These images were performed in 355 male and 322 female children born between 1928 and 1974, from the first month of life up to the age of 22 years (124). Data obtained in this study were introduced in a computerized system that analyzes 111 maturity indicators of the hand and wrist area in relation to sex and age, morphology, contiguity ratios, and through linear measurements of some bone segments (125, 126). For the Fels method, the prediction of adult height is calculated with the Roche–Wainer–Thissen formula. Although this method is very accurate and allows doctors to estimate children's bone age even when they are <1 year old, the Fels method is too complex, thus minimizing its daily use.

### Comparison of the Different Methods of Assessment of Bone Age

These methods differ according to the technique of the procedure and particularly to peculiar advantages and disadvantages (Table 1). The main aspects of these differences are summarized in Table 1 focused on variability, time of execution, radiation risk, and standardization. According to our experience in the field, the best approach might be the Greulich-Pyle (GP) method. However, the GP method requires a continuous and long experience in order to optimize bone age determination. Therefore, newer methods, such as artificial intelligence, might be used with the aim to guide endocrinologists and radiologists in the daily routine approach.

**Table 1:** Principal pros and cons related to the different methods utilized for the definition of skeletal age [82, 85, 94, 95, 97, 98, 102, 116, 121, 122, 123, 126, 129,132, 135,137, 138].

	**Method**	**Disadvantages**	**Advantages**	**Radiation risk**
GP	Visual inspection Correspondence method	Greater variability between observers compared to the TW method	Quick execution Used by more than 76% of pediatricians	Very low
TW	Visual and scoring method: the sum of scores reflects general skeletal development	Subjective evaluation of bone age. Takes time	More reliable than GP method	Very low
Fels	More reliable than GP method	Limited experience	Standardized evaluation of errors Useful for forensic use	Very low
Computerizedassisted techniques	Computerized calculation of bone age using wrist radiographs	Automated evaluation, but not totally eliminated radiologist and pediatrician evaluation	Accuracy Precision	Very low
Ultrasound	The technique uses growth cartilages dimensions in three orientations: front, back and side	Operator-dependent Difficulty of standardization Needs further improvements	Accessibility Quick scan Low cost Multiplanar capacity Comparison with contralateral	Absent

## Computerized Automatic Systems for Reading Bone Age: Potentials and Limits

In the last 20 years, newer methods have also been studied with the principal aim to mainly eliminate the variability related to interpretation according to the different methods. In 1991, Pietka et al. (127) started a computerization project of reading using phalanxes length compared to atlas. This system allows the computer to perform reading operations. Furthermore, the image was digitized and transformed into a series of mathematical coefficients produced mostly from standard image of the left hand and wrist X-rays. Since then, more than 15 new computerized automatic systems have been developed (128, 129). These systems use different algorithms; thus, no standardized and universally accepted indexes have been proposed so far (130, 131). In 2008, a new fully automated system was introduced, known as BoneXpert (Visiana, Denmark), with a reading time between 1.5 and 4 min. This system does not take into account the state of carpus bones maturation and allows a bone age assessment between 2.5 and 17 years and 2.0 and 15.0 years for male and female individuals, respectively. This software is validated for different ethnic groups and for children with different endocrine disorders (132–134). According to a recent study, the BoneXpert method is affected by obesity to a lesser extent than the Greulich–Pyle method. However, this system has some limits that must be considered, in particular, the absence of carpal bones evaluation, the opposition of local administrations to install the software, and the higher cost compared to available methods (GP and TW) (134).

In our opinion, this method could be useful also to obtain information about:

- defects in condrogenesis and/or osteogenesis (commonly found in hypochondroplasia);
- irregularity of metaphyseal regions and enlargement of the metaphyseal region of the ulna and of the radius (commonly found in subjects with rickets or metaphyseal chondrodysplasias);
- shortening of the fourth metacarpus, triangularization of radius distal epiphysis, pyramidalization of carpus distal line, or translucency of radius (commonly found in Leri–Weil and Turner Syndrome);
- shortening of the fourth and fifth metacarpus (commonly found in pseudohypoparathyroidism);
- Harris lines (expression of a temporary arrest of long bones growth); and
- defects in bone mineralization process (commonly found in osteochondrodysplasia).

Moreover, according to recent studies, BoneXpert computer-automated bone age determination method showed a significant positive correlation with chronological age and Greulich–Pyle. Furthermore, the impact of being overweight or obese on bone age could be identified correctly by BoneExpert. For these reasons, BoneExpert is considered a valid method.

## Ultrasound

Over the years, practitioners have tried to assess bone age by ultrasound. Among the different procedures proposed, BonAge system represents an ultrasound machine that includes a probe connected to a main unit. The probe is made up of two portions: the first one that emits radiofrequencies (750 kHz) that are directed against the surface of ulna and the radio epithelium and the second probe that receives radiofrequencies. The entire procedure takes about 5 min during which 11 measuring cycles are performed. Then, the final report is done, making an average of the measurements. It is based on a computerized system obtained from a series of measurements provided by a large reference population.

Although encouraging results have been shown, this method still requires improvements in terms of reproducibility and elimination of confounding factors (135, 136).

## Height Prediction

Several authors have proposed different algorithms for predicting adult height. Among them, the most used is based on the tables developed by Bayley and Pinneau in 1946 and revised in 1959. These tables have been formulated on bone age assessment according to the standards of Greulich and Pyle. Particularly, the tables are based on the assumption that there is a correlation between the proportion of adult stature reached at that time and skeletal age.

The Bayley–Pinneau method uses a series of tables that are indexed according to gender, chronological age, and skeletal age. Tables are provided for ages 7–18 years. This system of prediction is based on the fact that skeletal age correlates with a specific percentage of mature height reached in a specific moment when chronological age is constant. Although useful and easy to use, this method might be affected by several causes of errors. In particular, these prediction tables are developed from the Greulich–Pyle standards for hands, thus with the expectation that they will be used in conjunction with these standards (137, 138).

Another method is the Roche–Wainer–Thissen (RWT) algorithm, which calculates predicted adult height directly from a linear combination of the child's weight, recumbent length, and bone age, together with parental height, by using a gender- and age-specific coefficients. Coefficients used in the RWT method are tabulated to 14 years of age for girls and 16 years of age for boys (138). According to a recent study, the BP method predicts lower adult heights than the RWT method (139). In another study on 62 boys and 28 girls with short stature, BP method is more accurate for short boys than short girls (140).

It is important to highlight that all the available methods might be carefully used in the daily clinical approach in order to avoid unreliable expectation in children and parents. For example, according to Martin et al. (10), adult height may be overestimated in constitutional delay, and at the same time, it may be underestimated in idiopathic short stature.

To note, growth patterns may be influenced by relevant and common confounding factors and particularly illnesses, diet, and hormone imbalances.

As well, height prediction methods might be affected by ethnicity-related differences, thus either underestimating or overestimating adult height, with wide variations in accuracy.

Moreover, even when there is a good correlation between predicted and actual adult height, there is a wide individual variation, with almost 30% of adults differing by more than 5.0 cm from the BP predicted height (141).

For this reason, pediatricians should evaluate each prediction of future height on the bases of all the available knowledge about the child, particularly their personal growth history.

## Relevance of the Variability Related to the Operator and Between the Operators

Using an atlas-based method gives a great possibility of intra- and interoperator variability (142). As known, operator variability (intravariability) is defined by the degree of variability in the interpretation of same data performed at two different times by the same operator. Instead, the variability among different operators (intervariability) is defined by the degree of variability in the interpretation of same data made by two different operators at the same time. In order to achieve a greater accuracy and diagnostic reproducibility, it is important that bone age determination has the lowest intra- and interoperator variability. The GP and TW methods are characterized by a considerable variability. Thus, some authors suggest that, whenever possible, the same method should be used, favoring TW2 method if possible (93).

In a study conducted by King and collaborators in which bone aging was performed by three different operators using either GP or TW method, there was a significant intraindividual variability with values equal to 0.96 and 0.74 years, respectively (94). Furthermore, the GP method has not been updated from its initial publication, representing important limits of applications especially in some ethnic groups such as African or Hispanic female subjects and in Asian and Hispanic male subjects during late infancy and adolescence (95, 96). In fact, in the beginning, data were obtained from Caucasian children, so it is easy to understand that results assessed by the GP and TW standards are strongly dependent on ethnic group.

It was documented that GP standards are highly inaccurate in children born in America from African or European parents (84). In another study, it was shown that the evaluation of 599 bone age in subjects belonging to different ethnic groups shows a greater variability, especially in African children, in Hispanic women and in Asian and American men (96).

The following are generally documented (84, 97–105):

- a delay in bone age in Middle-Eastern men and Iranian men and in Southeast Asian children (Indonesian and Indian men and women) and Asian-American men;
- an advanced age in Afro-American children (more in female than in male) and in Middle Eastern female children (Iranian girls);
- no variation is documented for Italian, Korean, and Scottish children and in Pakistani girls.

## Conclusions

Currently, hand and wrist X-ray is the gold standard to assess children's bone age. Regardless of the method used, an appropriate and standardized hand positioning procedure and radiographic image acquisition are required in order to better describe the skeletal maturation. Over the years, many standardized methods have been developed to evaluate a skeletal maturity score for hand and wrist X-rays. Among these, three methods are the most representative: Greulich–Pyle method, Tanner–Whitehouse method, and Fels method. Using an atlas-based method gives a great possibility of intra- and interoperator variability, so in the last 20 years, new methods have been studied such as computerized automatic systems. Assessing bone age is also important to predict adult height. There are different methods to predict adult height, but the most used are the BayleyPinneau method and Roche–Wainer–Thissen method.

To note, a proper assessment of bone age must always take into account differences between ethnic groups, sex, and any present pathological conditions. For this reason, pediatricians should evaluate patients on the bases of all the available knowledge about the child, particularly their personal growth history.

## Author Contributions

FCa, CG, AM, and FCh have contributed to the conception and the design of the manuscript. CG has organized the material. FCa has written the first draft of the manuscript. CG has written sections of the manuscript. All authors contributed to manuscript revision and read and approved the submitted version.

## Conflict of Interest

The authors declare that the research was conducted in the absence of any commercial or financial relationships that could be construed as a potential conflict of interest.
